# Standing balance therapy through portable and low-cost visual feedback training

**DOI:** 10.1186/s12938-025-01507-0

**Published:** 2026-01-15

**Authors:** Mohammad Shushtari, William Pei, Derrick Lim, Kei Masani

**Affiliations:** 1 Institute of Biomedical Engineering, University of Toronto, 172 St. George St., Toronto, Ontario M5R 0A3 Canada; 2 KITE Research Institute, University Health Network, 520 Sutherland Drive, Toronto, Ontario M4G 3V9 Canada; 3 Department of Mechanical, Industrial, and Mechatronics Engineering, Toronto Metropolitan University, 87 Gerrard St. East, Toronto, Ontario M5B 2K3 Canada

**Keywords:** Balance, Visual feedback training, Rehabilitation

## Abstract

Individuals with incomplete spinal cord injury (iSCI) often fall due to decreased sensorimotor integration. Functional electrical stimulation (FES) therapy combined with visual feedback balance training (VFBT), termed FES+VFBT, can effectively improve standing balance in iSCI populations. Although promising, the need for force plates (FP), which are expensive and bulky, limits the translation of these methods to clinical and home settings. In this work, we propose a solution by replacing FP with Wii Balance Board (WBB), allowing for more accessible FES+VFBT at a lower cost in both clinical and community settings. Our investigations on ten non-injured participants reveal that WBB-based estimated center of mass (COM) has low prediction error and high correlation in both anteroposterior (RMSE: 4.13 ± 0.69 mm, *r*: 0.94 ± 0.02) and mediolateral directions (RMSE: 6.25 ± 1.80 mm, *r*: 0.92 ± 0.04) with ground FP-estimated COM, resulting in similar stimulation patterns obtained with the WBB-based approach, indicating that the WBB-based FES+VFBT system could yield a more accessible therapeutic strategy for balance rehabilitation in iSCI.

## Introduction

Spinal cord injury (SCI) results in a wide range of sensory and motor impairments below the injury level. In particular, incomplete spinal cord injury (iSCI) is characterized by partial preservation of motor and sensory functions, often permitting some degree of functional recovery including the ability to regain walking ability. Despite these gains, sensorimotor deficits significantly impair balance control, greatly increasing the risk of falls in individuals with iSCI [1]. Epidemiological data indicate a high prevalence of falls, with reports suggesting that up to 78% of individuals with iSCI experience at least one fall annually [2]. These falls have substantial health consequences including fractures, soft tissue injuries, hospitalizations, and psychological distress, and contribute significantly to healthcare expenditures—estimated to add approximately $10 billion annually to the Canadian healthcare system alone [3]. Effective balance training is therefore critical not only for reducing fall risk and enhancing independence, but also for significantly decreasing the associated economic burden [4].

Despite the recognized importance of balance training, its implementation in inpatient rehabilitation remains limited, averaging merely $2.2\pm 2.4$ hours over an entire rehabilitation stay [5]. Current rehabilitation strategies tend to prioritize muscle strengthening and reactive balance responses, frequently overlooking sensory integration and proactive balance control mechanisms [6–9]. Consequently, there is a critical need for balance interventions that effectively target sensorimotor integration deficits, a hallmark challenge in the rehabilitation of individuals with neurological impairments such as iSCI.

Balance control inherently depends on the intricate integration of somatosensory, visual, and vestibular systems. Individuals with iSCI are often disproportionately reliant on visual inputs for maintaining postural stability compared to able-bodied individuals [8, 10]. This greater reliance on vision has catalyzed research interest in visual feedback balance training (VFBT), a modality in which participants receive real-time visual feedback of their center of pressure (COP) position on a monitor. Through VFBT, participants actively adjust their postural sway to follow visual targets, thereby directly enhancing sensorimotor integration. Studies have reported that VFBT significantly improves standing balance performance in neurological populations, including SCI and stroke survivors, with improvements in COP sway metrics ranging from 10% to 70% depending on the specific outcome measure employed [8, 9, 11, 12].

Overemphasizing on VFBT for balance rehabilitation can, however, result in developing dependency on visual cues for balance and, therefore, worsen the balance control after training period is over when no visual cues are provided [13]. To further enhance the therapeutic efficacy of balance interventions, our research group previously developed an innovative system that integrates VFBT with closed-loop functional electrical stimulation (FES), termed FES+VFBT [14–18]. FES facilitates active muscle contractions by applying controlled electrical currents through transcutaneous electrodes to ankle dorsiflexors and plantarflexors, thus enabling individuals with impaired voluntary muscle control to engage actively in targeted balance training. This intervention has shown promise in pilot studies involving individuals with iSCI, demonstrating increased COP displacement ranges, enhanced standing stability, and improved balance confidence [19, 20].

Despite its promising therapeutic outcomes, the existing FES+VFBT setup depends heavily on laboratory-grade force plates (FP) resulting in high costs and substantial practical limitations that hinder clinical translation and broader community access [15–18]. Traditionally, FPs represent the gold standard for measuring COP due to their precision and reliability. However, their widespread clinical implementation remains constrained due to their high cost, considerable size, technical complexity, and limited portability.

The expensive nature and complexity of laboratory-grade FPs exemplify the persistent translational gap between research innovations and practical clinical applications, frequently described as the “valley of death” [21]. Bridging this gap necessitates developing affordable, portable, and user-friendly alternatives suitable for widespread clinical deployment and home use [15–18, 22, 23]. The commercially available Wii Balance Board (WBB) serves as a compelling proof-of-concept for this approach. While the device itself is discontinued, its simple hardware—consisting of four vertical force sensors, a small CPU, and Bluetooth communication—demonstrates that accurate and reliable center-of-pressure measurements can be achieved with low-cost, readily available components. Although it lacks an official software development kit (SDK), the hobbyist community successfully reverse-engineered its communication protocol, highlighting the straightforward nature of interfacing with such devices. Extensive validation studies have confirmed that the WBB measures COP with accuracy comparable to standard laboratory FPs, maintaining consistent reliability across various users and prolonged usage periods [24–29]. The success of the WBB in research settings underscores the potential of integrating similar low-cost, custom-built force-sensing systems within an FES+VFBT framework, holding substantial promise for clinical translation, community rehabilitation, and home-based balance therapy.

In this study, we propose to replace the conventional FP with the WBB in our previously developed FES+VFBT system. While the WBB’s efficacy for static balance assessment is well-established, its capability to drive real-time, closed-loop FES systems remains unexplored. This distinction is critical, as closed-loop control requires not only accurate measurement but also low latency and signal stability to prevent erroneous stimulation. Consequently, our primary aim is to rigorously validate the performance of the newly adapted WBB-FES+VFBT system against the established FP-based system. While these devices directly measure the COP, our system estimates the body’s center of mass (COM) from this data, as it provides a more direct measure of the body’s postural sway for controlling the FES. We hypothesize that the WBB-based system will provide statistically equivalent performance metrics—specifically regarding the generated stimulation commands—compared to the FP-based system, thereby demonstrating its feasibility and paving the way toward more accessible and cost-effective clinical translation for balance rehabilitation in individuals with iSCI.

In this study, we propose to replace the conventional FP with the WBB in our previously developed FES+VFBT system. Our primary aim is to rigorously validate the performance of the newly adapted WBB-FES+VFBT system against the established FP-based system. While these devices directly measure the COP, our system estimates the body’s center of mass (COM) from this data, as it provides a more direct measure of the body’s postural sway for controlling the FES. We hypothesize that the WBB-based system will provide statistically equivalent performance metrics compared to the FP-based system, thereby demonstrating its feasibility and paving the way toward more accessible and cost-effective clinical translation for balance rehabilitation in individuals with iSCI.

**Fig. 1:**
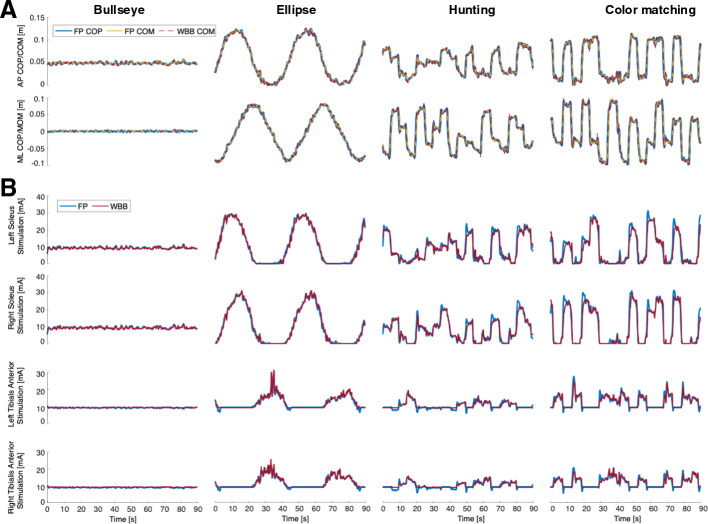
Time series from an example participant showing the measured center of pressure (COP) and estimated center of mass (COM) using the force plate (FP) and the Wii Balance Board (WBB) (**A**), along with the commanded functional electrical stimulation (FES) to the soleus and tibialis anterior muscles of both left and right legs (**B**) during the Bullseye, Ellipse, Hunting, and Color Matching games

## Results

### Verification of WBB COP and estimated COM

**Fig. 2:**
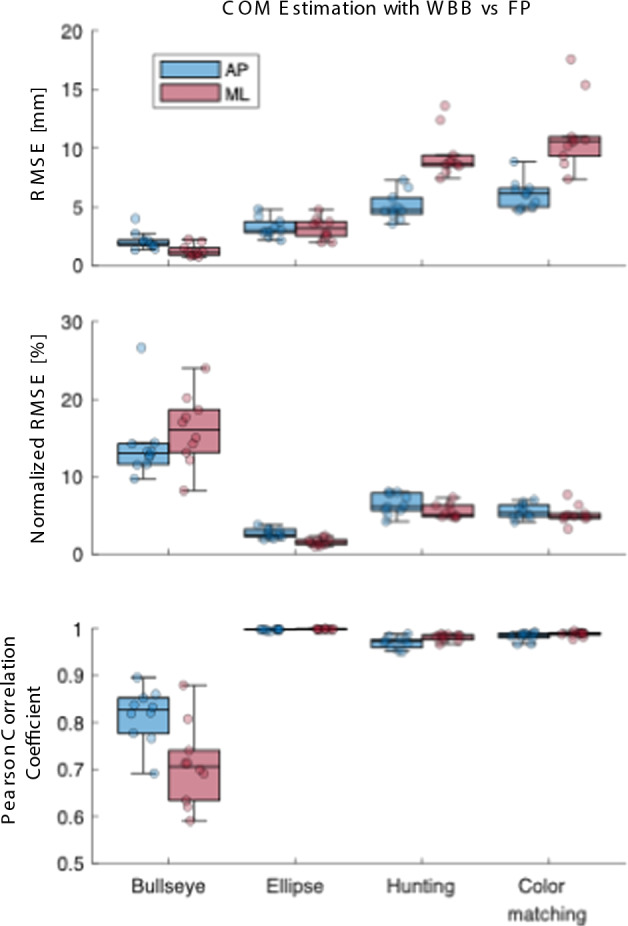
Boxplots showing the center of mass (COM) estimation accuracy using the Wii Balance Board (WBB) compared to the force plate (FP) across four visual feedback balance training (VFBT) tasks: Bullseye, Ellipse, Hunting, and Color Matching. Metrics include root mean squared error (RMSE) in millimeters, normalized RMSE (NRMSE) as a percentage of peak-to-peak range, and Pearson correlation coefficient between the systems. Results are shown separately for anterior–posterior (AP, blue) and mediolateral (ML, red) directions. In these plots, lower values for RMSE/NRMSE and higher values for correlation indicate better agreement between the WBB and FP systems

Figure 1A depicts examples of COP/COM across various VFBT tasks, demonstrating strong alignment between the WBB and FP measurements. Figure 2 and Table 1 also summarize the quantitative analysis. To aid interpretation, we utilized root mean squared error (RMSE) to quantify the average magnitude of error (where values closer to 0 mm indicate better accuracy) and Pearson’s correlation coefficient (*r*) to assess signal similarity (where values closer to 1.0 indicate a strong match). We also calculated normalized RMSE (NRMSE) to express the error as a percentage of the movement range. The analysis reveals that the Bullseye task had the lowest error (ML: $1.25\pm 0.49$ mm, AP: $2.11\pm 0.74$ mm) due to its smaller dynamic range. Notably, the hunting and color matching tasks exhibited slightly greater RMSE when comparing WBB-based estimated COM with the FP-based estimated COM due to rapid COM excursions, despite the ellipse task having the largest dynamic range which highlights the need for a normalization. Normalized RMSE illustrates low NRMSE values for dynamic tasks when comparing WBB-based estimated COM against FP-based estimated COM (ML: NRMSE $<4.11\pm 0.85\%$, AP: NRMSE $<4.9\pm 0.95\%$) averaged across dynamic tasks. The NRMSE is, however, relatively higher for the Bullseye task. The small RMSE obtained for this task, nevertheless, indicates that the larger NRMSE is due to the smaller dynamic range rather than estimation accuracy. Pearson correlation analysis further highlights the accuracy of the WBB-estimated COM showing a strong match WBB-estimated COM and FP-based COM (ML: $r>0.98$, AP: $r>0.97$) across the dynamic exercises, although correlations with FP-estimated COM were slightly lower, especially for the Bullseye task (ML: $r:0.71\pm 0.08$, AP: $0.82\pm 0.05$) which is expected due to the small dynamic range. These analyses collectively indicate that the WBB provides accurate COP measurements and a reliable representation of postural movements during VFBT tasks.

**Fig. 3:**
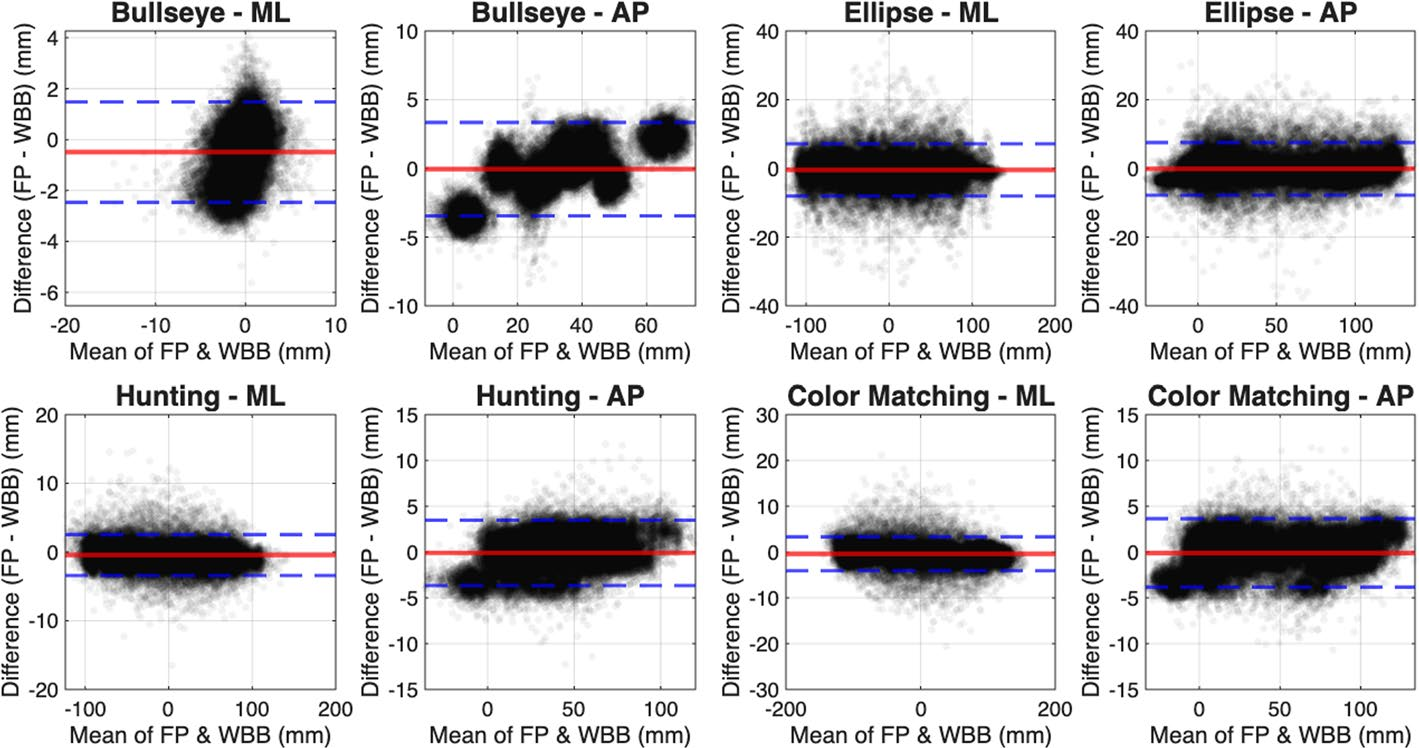
Bland–Altman plots comparing the center-of-pressure (COP) measurements between the force plate (FP) and Wii Balance Board (WBB) across all four tasks. The red solid line represents the mean difference (Bias), which is near zero for all conditions, indicating no systematic error. The blue dashed lines represent the 95% limits of agreement ($\pm 1.96$ SD). Results are separated by mediolateral (ML) and anteroposterior (AP) directions

To further rigorously assess the agreement between the two measurement systems, we performed a Bland–Altman analysis on the raw COP time-series data (Fig. 3). The analysis revealed a negligible systematic bias across all tasks and directions, with mean differences ranging from $-0.05$ mm to $-0.49$ mm. This indicates that the WBB does not consistently over- or underestimate postural sway compared to the reference force plate. The 95% limits of agreement (LoA) were narrowest for the static Bullseye task (ML: $-\,2.47$ to 1.48 mm; AP: $-\,3.45$ to 3.34 mm) and widest for the large-excursion Ellipse task (ML: $-\,8.07$ to 7.17 mm; AP: $-\,7.79$ to 7.51 mm). However, even in these dynamic tasks, the random error remained small relative to the total sway amplitude, supporting the claim of statistical equivalence.

**Table 1:** COM estimation accuracy using the Wii Balance Board (WBB) compared to the FP, across four visual feedback balance training (VFBT) tasks

		Bullseye	Ellipse	Hunting	Color Matching
COM AP	RMSE [mm]	$2.11\pm 0.74$	$3.23\pm 0.77$	$5.06\pm 1.11$	$6.10\pm 1.17$
	NRMSE [%]	$14.00\pm 4.41$	$2.70\pm 0.57$	$6.44\pm 1.26$	$5.57\pm 0.90$
	Pearson Corr	$0.82\pm 0.05$	$1.00\pm 0.00$	$0.97\pm 0.01$	$0.98\pm 0.01$
COM ML	RMSE [mm]	$1.25\pm 0.49$	$3.15\pm 0.87$	$9.44\pm 1.87$	$11.14\pm 2.91$
	NRMSE [%]	$16.02\pm 4.24$	$1.64\pm 0.46$	$5.53\pm 0.84$	$5.15\pm 1.11$
	Pearson Corr	$0.71\pm 0.08$	$1.00\pm 0.00$	$0.98\pm 0.01$	$0.99\pm 0.00$

Metrics include root mean squared error (RMSE), normalized RMSE (NRMSE), and Pearson correlation coefficients for both anterior–posterior (AP) and mediolateral (ML) directions. Values are reported as mean ± standard deviation across participants

### Clinical vs. lab-based stimulation command

**Fig. 4:**
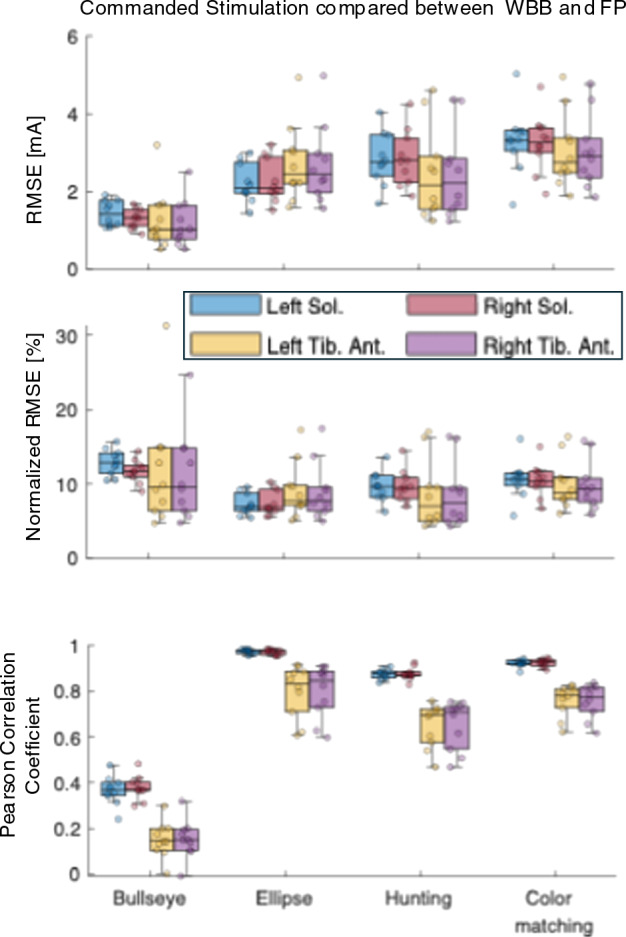
Boxplots comparing the commanded functional electrical stimulation (FES) between the Wii Balance Board (WBB)-based system and the force plate (FP)-based system across four visual feedback balance training (VFBT) tasks. Shown are the root mean squared error (RMSE), normalized RMSE (NRMSE), and Pearson correlation coefficient of the stimulation commands for the soleus (Sol.) and tibialis anterior (Tib. Ant.) muscles in both left and right legs. Lower error bars and higher correlation coefficients demonstrate closer agreement between the clinical (WBB) and lab-based (FP) stimulation commands

Similarly, Figure 1B shows timeseries of the stimulations for the soleus and tibialis anterior of both legs commanded by the original (FP-based) and the clinical (WBB-based) systems indicating that the close match between the estimation COMs has also led to a close match between the commanded stimulation in both systems. Figure 4 provides a summary of the comparison between the commanded stimulations across all four exercises and Table 2 further provides quantitative results of such comparison. RMSE results indicated consistency between the commanded stimulation across tasks, with slightly lower errors for the Bullseye task (SOL: RMSE $1.37\pm 0.28$ mA, TA: RMSE $1.24\pm 0.66$ mA) compared to dynamic tasks (SOL: RMSE $2.79\pm 0.64$ mA, TA: RMSE $2.74\pm 0.96$ mA). The NRMSE demonstrated a higher error for the Bullseye task due to its smaller dynamic range. Despite lower correlations for TA, its absolute accuracy remained comparable to soleus across all tasks. The Pearson correlation between the stimulation commands during dynamic tasks (ellipse, hunting, color matching) displays higher correlations (SOL: $r>0.87$, TA: $r>0.65$) compared to the Bullseye task (SOL: $r>0.36$, TA: $r>0.15$), primarily due to minimal stimulation fluctuations during the Bullseye task which means the Pearson correlation during the Bullseye task is affected more by the signal noise rather than the actual variations of the COM. Additionally, the tibialis anterior correlations were consistently lower due to its reduced modulation range relative to the soleus.

## Discussion

### Stimulation control accuracy

Figure 1B shows that in all VFBT tasks, the stimulation command from the lab-based (FP-based) system has slightly higher noise compared to the clinical system (WBB-based). This likely originates from the high sensitivity of the FP to the participant’s micro-movements. These fluctuations, amplified by the derivative action of the FES controller, contributes to the reduction of the correlation values compared to the WBB system (Table 2). Filtering to remove such fluctuations would introduce unwanted delay in real-time FES control. Moreover, the arbitrary and relatively large dynamic range between motor threshold and maximum tolerable stimulation used in this study amplified sensitivity to these fluctuations. Despite this limitation, the WBB system effectively replicated the stimulation trends of the lab-based system.

This reliability is further supported by the Bland–Altman analysis, which demonstrated a near-zero bias across all exercises. This finding indicates that the WBB does not introduce systematic drift or offset errors, which is critical for maintaining a stable control setpoint during long-duration balance training. While the limits of agreement were wider for dynamic tasks compared to static standing, this proportional increase in variance is expected with larger rapid excursions. Crucially, these random errors remained small relative to the user’s total range of motion and did not negatively impact the FES controller’s ability to generate appropriate stimulation commands, as evidenced by the high fidelity of the stimulation profiles.

**Table 2:** Comparison of functional electrical stimulation (FES) commands generated by the WBB-based (clinical) system and the FP-based (lab) system across all muscles and task

		Bullseye	Ellipse	Hunting	Color Matching
Left Sol	RMSE [mA]	$1.44\pm 0.31$	$2.22\pm 0.48$	$2.84\pm 0.66$	$3.30\pm 0.80$
	NRMSE [%]	$12.91\pm 1.67$	$7.26\pm 1.38$	$9.65\pm 1.91$	$10.64\pm 2.45$
	Pearson Corr	$0.36\pm 0.06$	$0.97\pm 0.01$	$0.87\pm 0.02$	$0.92\pm 0.02$
Right Sol	RMSE [mA]	$1.30\pm 0.24$	$2.27\pm 0.53$	$2.86\pm 0.69$	$3.24\pm 0.71$
	NRMSE [%]	$11.71\pm 1.47$	$7.42\pm 1.57$	$9.72\pm 2.05$	$10.46\pm 2.15$
	Pearson Corr	$0.38\pm 0.05$	$0.97\pm 0.01$	$0.88\pm 0.03$	$0.92\pm 0.02$
Left TA	RMSE [mA]	$1.27\pm 0.74$	$2.73\pm 0.94$	$2.46\pm 1.13$	$3.06\pm 0.91$
	NRMSE [%]	$11.75\pm 7.37$	$9.10\pm 3.59$	$8.62\pm 4.42$	$9.97\pm 3.23$
	Pearson Corr	$0.15\pm 0.07$	$0.80\pm 0.11$	$0.65\pm 0.09$	$0.76\pm 0.07$
Right TA	RMSE [mA]	$1.20\pm 0.57$	$2.69\pm 0.96$	$2.46\pm 1.08$	$3.02\pm 0.88$
	NRMSE [%]	$11.08\pm 5.69$	$8.98\pm 3.67$	$8.61\pm 4.24$	$9.87\pm 3.17$
	Pearson Corr	$0.15\pm 0.08$	$0.80\pm 0.11$	$0.65\pm 0.10$	$0.76\pm 0.07$

Shown are root mean squared error (RMSE), normalized RMSE (NRMSE), and Pearson correlation coefficients for left/right soleus and tibialis anterior (TA) muscles during each of the four VFBT tasks

### Clinical system practicality

Unlike previous studies that solely validated the WBB for passive balance assessment, this work establishes its viability as an active sensor in a closed-loop therapeutic system. The critical finding is that the WBB’s lower sampling rate and resolution did not compromise the stability or accuracy of the FES controller. The clinical system accurately replicated lab-based stimulation patterns across dynamic tasks, reflected by high correlation and consistent error metrics. Lower correlations during minimal movement tasks (Bullseye) were expected and did not reflect decreased accuracy. Despite differences in correlation values, absolute stimulation accuracy remained consistent, underscoring the clinical system’s reliability. While this study focused on concurrent validity, the test–retest reliability of the WBB hardware has been extensively established in prior literature [14, 27, 28]. To ensure this reliability translates to consistent FES control across days, our system incorporates a mandatory calibration routine (quiet standing and limits of stability) at the start of every session, normalizing the controller to the user’s daily baseline.

From a practical implementation standpoint, this system offers a streamlined workflow for real-world rehabilitation. The Bluetooth connectivity eliminates complex wiring, reducing setup time compared to wired force plates. Furthermore, the Unity interface abstracts the complex control parameters, allowing therapists to focus on patient safety and therapeutic progression—such as modulating game difficulty and visual targets—rather than managing signal acquisition. By automating the calibration and COM estimation steps, the system is designed to be operated with minimal technical training, facilitating its integration into busy outpatient clinics or potential home-based tele-rehabilitation scenarios. The feasibility of this workflow is currently being demonstrated in an ongoing clinical trial, where the system is successfully operated by clinical staff independent of the engineering team.

### Limitations

A key limitation is that the WBB cannot directly measure COM, necessitating an extra estimation step to enable COM-based control. Although this limitation did not significantly impact stimulation control performance, future studies could explore enhanced methods for indirect COM estimation. Moreover, the WBB’s elevation could pose practical challenges for individuals with impaired balance, potentially increasing the risk of falls.

The transition from healthy validation to clinical application in iSCI requires specific adaptations to address altered postural control mechanisms. First, individuals with iSCI often exhibit compromised ankle control and may utilize a hip strategy to maintain balance. Since our current COM estimation relies on an inverted pendulum model—which assumes an ankle strategy—future iterations may need to incorporate multi-segmental models or additional inertial sensors to improve estimation accuracy in patients with significant hip flexion. Accordingly, future developments should also explore expanding the FES protocol to include proximal lower limb and trunk muscles, which would provide more comprehensive control for individuals relying on hip-based balance strategies. Second, spasticity and clonus are common in iSCI. Our results indicated that the WBB effectively acts as a low-pass filter, producing smoother stimulation commands than the highly sensitive FP. This characteristic may be advantageous in filtering out high-frequency tremors associated with spasticity. However, clinical tuning will likely be necessary; specifically, the "dead zone" and gain of the FES controller may need adjustment to prevent the system from reacting to non-functional spastic movements while still responding to genuine postural sway. Furthermore, to mitigate fall risks associated with the WBB’s elevation, future clinical setups must integrate safety measures such as an overhead safety harness, parallel bars, or a custom platform surround to create a flush surface with the device.

Finally, regarding the sustainability of this solution, we acknowledge that the WBB hardware has been officially discontinued. However, due to the massive volume of units produced, it remains widely available and affordable on the secondary market (<$50 USD), ensuring immediate accessibility for clinical uptake. More importantly, this study serves as a proof-of-concept that low-cost, consumer-grade force sensors with limited sampling rates (approx. 50-100Hz) and resolution are sufficient for closed-loop FES control. Consequently, the system is not strictly bound to the WBB; modern alternatives, such as open-source custom load cell arrays or generic Bluetooth-enabled balance scales with comparable specifications, can be readily integrated into the developed Unity framework to ensure long-term clinical viability.

## Conclusion

This study successfully demonstrated the viability of a clinically adapted FES+VFBT system using the Unity game engine, significantly improving user-friendliness and reducing redundancy. The WBB effectively measures COP, closely matching the FP’s performance. Utilizing an estimated COM from the WBB as a controller input produced stimulation patterns comparable to the gold standard FP-based system, affirming the clinical system’s practicality. These findings represent a substantial advancement toward bringing standing balance therapy into clinical practice.

## Materials and methods

**Fig. 5:**
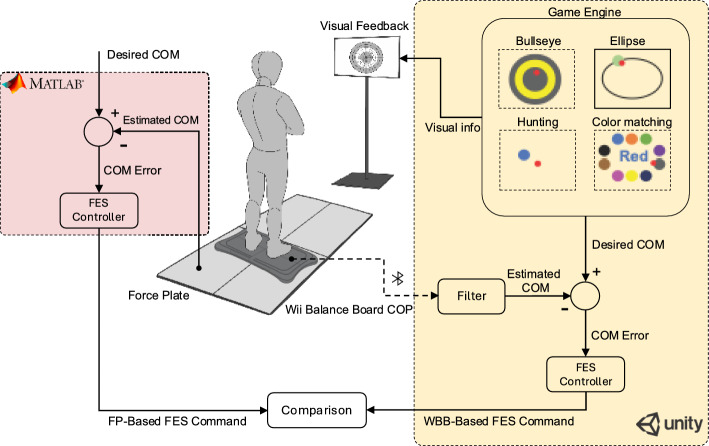
Overview of the comparison of the clinical (WBB-based) and lab-based (FP-based) VFBT systems implemented in Unity and Matlab, respectively. Both systems work based on COM feedback from either FP or WBB which is then used to obtain the COM error with respect to a desired COM location determined according to a game engine implemented in Unity. COM error is then fed into a PD controller (similarly implemented in Matlab and Unity) which generates an FES command. Commanded FES and the obtained COM from both methods are then compared. Each user completes four different games (Bullseye, Ellipse, Hunting, and Color matching) for three times while receiving visual feedback

### Clinical system development

The original FES + VFBT system utilizes a force plate (AccuSway-Dual, AMTI, Watertown, USA) to estimate the center of mass (COM) based on the measured ground reaction forces and moments. Estimated COM is used for visual feedback and to provide input to a physiologically inspired controller to determine the required ankle torque for balance. An appropriate electrical stimulation is calculated to realize the desired ankle torque by stimulating the soleus and tibialis anterior muscles of each leg according to [14]. To develop a clinical version of this system, we replaced the FP with a Wii Balance Board (WBB; Nintendo, Japan). The WBB contains four strain-gauge sensors, an internal CPU, analog-to-digital converters, and Bluetooth connectivity. The selection of the WBB was motivated by extensive literature demonstrating its performance is comparable to laboratory-grade FPs, establishing its potential as a reliable and cost-effective alternative [15, 17, 18, 23, 28, 29]. Bluetooth connectivity simplifies setup by eliminating additional wiring and acquisition hardware. Unity (Unity Technologies, USA) was selected as the development platform using the C# programming language. Unity streamlined graphical rendering, game physics, controller computations, and interface design, allowing straightforward modifications to accommodate clinical requirements. Existing C# libraries (Wiimotelib[30], WiiBuddy API [31]) further facilitated data extraction from the WBB.

**Table 3:** Summary of participant characteristics

Participant	Sex (M/F)	Age (years)	Height (cm)	Weight (kg)
P1	M	28	185	80
P2	M	26	178	73
P3	M	25	178	67
P4	M	25	178	67
P5	F	23	167	55
P6	F	35	165	50
P7	F	27	170	70
P8	F	23	160	54
P9	M	29	173	86
P10	F	26	180	67
Mean	–	26.7	173	66.9
STD	–	3.5	8	11.4

### Experimental validation

To validate the clinical system, ten non-injured participants (5 male, 5 female; mean age 26.7 ± 3.5 years; height 173 ± 8 cm; mass 66.9 ± 11.4 kg) (see Table 3 without neurological conditions practiced VFBT while their COP were measured using both the FPs and the Wii balance board and their COM were estimated accordingly. Participants completed three sets of four 100-s randomized VFBT exercises shown in Fig. 5. The exercises included: (1) Bullseye, maintaining position at the screen center; (2) Ellipse, tracking a target moving along an elliptical path; (3) Hunting, where participants moved their cursor to random targets appearing on-screen; and (4) Color Matching, requiring participants to select the target matching the color described by text not the text color itself (similar to the Stroop test).

Participants stood upright, arms crossed, primarily using ankle movements to shift their balance. All of the visualizations were rendered using Unity according to the COM estimated by the Wii balance board while the FP data were simultaneously recorded at 2000 Hz using Cortex 3.1 software (Motion Analysis Corp., Rohnert Park, CA). The WBB was positioned centrally on FPs to ensure ground reaction forces are projected on both WBB and FP similarly. Participants stood with ankles 0.17 m apart and feet rotated outward by 14 degrees [32]. Before the experiment, the VFBT system was initially calibrated to each participant range of motion and balance control by performing quiet standing (eyes open and closed, 100 s each) and limits of stability assessment by leaning in eight directions (0$^\circ$, 45$^\circ$,...,315$^\circ$). This study was approved by the Research Ethics Board of the University Health Network in accordance with the Declaration of Helsinki on the use of human participants in experiments.

### Data processing

Clinical system data (WBB-based) were recorded at 50 Hz and downsampled to 20 Hz to match the lab-based system’s (FP-based) controller frequency. All processing was performed within Unity. Lab-based system data from FPs (sampled at 2000 Hz) underwent high-pass Butterworth filtering (cutoff 0.15 Hz) to remove drift, followed by a 10 ms (20 samples) moving-average filter for noise reduction. COM was calculated using the inverted pendulum method [33] and then used to compute the FES command as the control output. Data processing was done offline using MATLAB (R2022a, MathWorks, USA).

### Data analysis

The clinical system’s performance was validated by comparing its estimated COM with that of the lab-based system. We also compared the stimulation (FES) commands in both systems to ensure that discrepancies in the measured COP and estimated COM between the two systems do not lead to a considerable error in the applied muscle stimulation. In this regard, an arbitrary stimulation maximum of 31.25 mA and threshold of 9.375 mA were used as calibration parameters of the FES system. A median filter was also applied to remove spikes in stimulation output caused by rapid COM movements [22, 34, 35].

To compare COM, COP, and FES timeseries across the two systems, we used root mean squared error (RMSE) and Pearson correlation coefficient averaged across each exercise and participant. To account for the variable range of motion and balance control in each participant, we also computed the normalized RMSE (NRMSE) by dividing the obtained RMSE over the peak-to-peak range of COP or COM in each exercise for each participant. Correlations were computed separately for the soleus and tibialis anterior muscles. Tibialis anterior analysis was restricted to active stimulation periods. All analyses were performed using MATLAB.

## Data Availability

The datasets generated and/or analysed during the current study are available from the corresponding author on reasonable request.
